# Diagnosing Internal Herniation After Roux-en-Y Gastric Bypass Surgery: Literature Overview, Cadaver Study and the Added Value of 3D CT Angiography

**DOI:** 10.1007/s11695-018-3121-3

**Published:** 2018-02-05

**Authors:** Cornelis Klop, Laura N. Deden, Edo O. Aarts, Ignace M. C. Janssen, Milan E. J. Pijl, Anneline van den Ende, Bart P. L. Witteman, Gabie M. de Jong, Theo J. Aufenacker, Cornelis H. Slump, Frits J. Berends

**Affiliations:** 1 MIRA Institute for Biomedical Technology and Technical Medicine, University of Twente, Enschede, The Netherlands; 2 Department of Bariatric Surgery, Rijnstate Hospital and Vitalys Clinic, Wagnerlaan 55, 6815 AD Arnhem, The Netherlands; 3 Department of Radiology, Rijnstate Hospital, Arnhem, The Netherlands

**Keywords:** Roux-en-Y gastric bypass, Complication, Internal herniation, Mesenteric defect, Closure, Petersen’s space, Literature overview, Cadaver study, Computed tomography, Angiography

## Abstract

**Purpose:**

The purposes of the study are to outline the complexity of diagnosing internal herniation after Roux-en-Y gastric bypass (RYGB) surgery and to investigate the added value of computed tomography angiography (CTA) for diagnosing internal herniation.

**Materials and Methods:**

A cadaver study was performed to investigate the manifestations of internal hernias and mesenteric vascularization. Furthermore, a prospective, ethics approved study with retrospective interpretation was conducted. Ten patients, clinically suspected for internal herniation, were prospectively included. After informed consent was obtained, these subjects underwent abdominal CT examination, including additional arterial phase CTA. All subjects underwent diagnostic laparoscopy for suspected internal herniation. The CTA was used to create a 3D reconstruction of the mesenteric arteries and surgical staples (3D CTA). The 3D CTA was interpreted, taking into account the presence and type of internal hernia that was found upon laparoscopy.

**Results:**

Cadaveric analysis demonstrated the complexity of internal herniation. It also confirmed the expected changes in vascular structure and surgical staple arrangement in the presence of internal herniation. 3D CTA studies of the subjects with active internal hernias demonstrated remarkable differences when compared to control 3D CTA studies. The blood supply of herniated intestinal limbs in particular showed abnormal trajectories. Additionally, enteroenterostomy staple lines had migrated or altered orientation.

**Conclusion:**

3D CTA is a promising technique for diagnosing active internal hernias. Our findings suggest that for diagnosing internal hernias, focus should probably shift from routine abdominal CT examination towards the 3D assessment of the mesenteric vasculature and surgical staples.

**Electronic supplementary material:**

The online version of this article (10.1007/s11695-018-3121-3) contains supplementary material, which is available to authorized users.

## Introduction

**Fig. 1:**
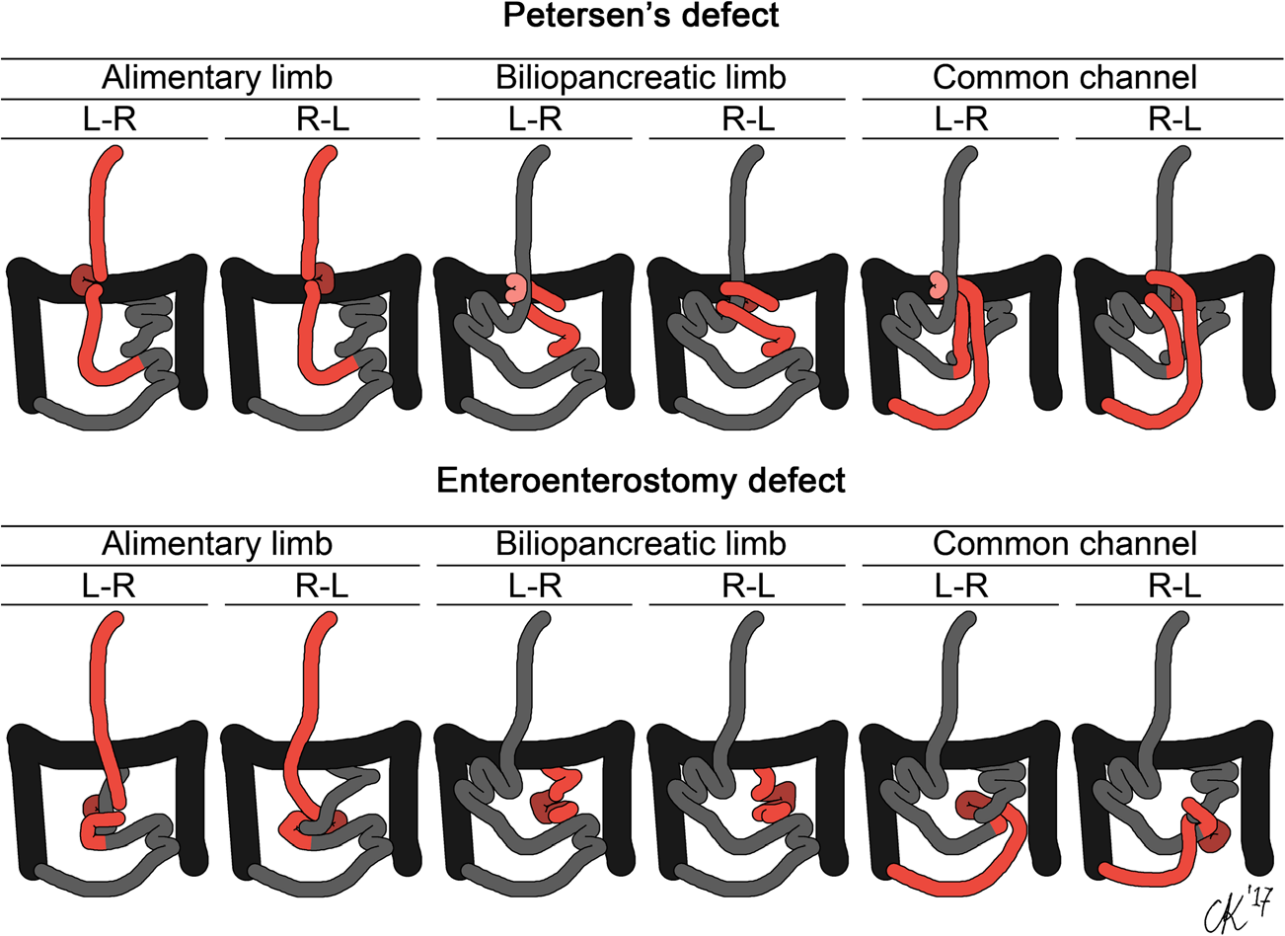
Schematic representations of all 12 theoretically possible internal hernias when a single mesenteric defect and intestinal limb are involved. L-R indicates a left-to-right hernia, R-L a right-to-left hernia

Worldwide, the laparoscopic Roux-en-Y gastric bypass (RYGB), is one of the most commonly performed bariatric procedures [1]. Although the advantages of bariatric surgery have been widely shown, many patients develop complaints that are related to the RYGB [2]. An important long-term complication is internal herniation, which is a common cause of small bowel obstruction (31–46%) after laparoscopic RYGB surgery [3–5]. Small bowel obstruction, in turn, is a serious complication, which may require emergent laparotomy to avoid bowel strangulation and infarction [3, 4]. Based on the mesenteric space, intestinal limb and herniation direction, there could in theory be at least 12 different identifiable internal hernia types (Fig. 1) [2]. Primary closure of the mesenteric defects with both staples and sutures has been proven effective for decreasing the incidence of internal herniation [6, 7].

Computed tomography (CT) examination is currently the first-line imaging technique used when there is a suspicion of internal herniation [8]. For many years, the swirl sign has been the best single indicator of an internal hernia after RYGB surgery. The swirl sign has a sensitivity of 39–100%; specificity ranges from 63 to 100% [9–14], thus indicating that CT examination is frequently inaccurate or inconclusive.

Despite being a worldwide problem, improving the current diagnostic tools is not a prevalent area of study. An alternative is not to consider the small intestine itself, but to investigate its blood supply. The mesenteric vascularization may appear stretched, crowded, engorged, or twisted when internal herniation is present, as a consequence of intestinal limb migration [15, 16]. Hence, abdominal CT angiography (CTA) may aid in the diagnosis of internal herniation. The present study investigates CTA for the diagnosis of internal herniation after RYGB surgery. In addition, a cadaver study was performed to support the presented hypothesis and to study the manifestations of internal hernias. An overview has also been provided to show the current status of diagnosing internal herniation.

## Materials and Methods

### Study Outline

The study consists of two parts. Firstly, the mesenteric vasculature and the manifestations of internal hernias were studied by means of a cadaver study. The second part is comprised of a clinical study, in which CTA was investigated for diagnosing internal herniation.

### Cadaver Study

RYGB surgery was performed on a cadaver in a dissection room. Informed consent was provided according to the will of the donor, who had dedicated their cadaver to human research studies. Both potential hernia were identified and all basic internal hernia types (Fig. 1) were simulated on the cadaveric intestines. During dissection, each intestinal limb and its corresponding blood supply were investigated.

### Study Design

Between September 2016 and April 2017, patients were prospectively included at our specialized, large volume bariatric center (+/−1200 procedures/year). The study was approved by the national and local ethics committees (NL52257.091.15) and registered at clinicaltrials.gov (NCT03114761). The subjects that participated in the study were clinically suspected for an active internal hernia after RYGB surgery. Following receipt of written informed consent, subjects underwent abdominal CT examination, including additional arterial phase CTA. If indicated by a bariatric surgeon, a patient was subjected to laparoscopic exploration for suspected internal herniation. The outcome of laparoscopic surgery served as the gold standard for the diagnosis.

### Primary RYGB Procedure

All study subjects underwent a laparoscopic RYGB procedure, following the antecolic, antegastric approach [17]. Using an Echelon linear stapler (Ethicon, Johnson & Johnson, New Brunswick, New Jersey, USA), a gastric pouch is constructed. Subsequently, a biliopancreatic limb of approximately 150 cm and an alimentary limb of about 100 cm are measured using the hand-over-hand technique. The gastroenterostomy and enteroenterostomy are constructed using a linear stapler, combined with hand-sewn closure of the remaining defect using a running Vicryl suture (Ethicon, Johnson & Johnson, New Brunswick, New Jersey, USA). Since 2011, both mesenteric defects are routinely closed in our clinic employing an Endopath EMS multifeed stapler (Ethicon, Johnson & Johnson, New Brunswick, New Jersey, USA). Each mesenteric defect is closed with a double-layered staple line of approximately ten staples in total per defect.

### Study Population

Subjects underwent standard RYGB surgery at least a half year earlier and were clinically suspected for internal herniation by a bariatric surgeon. Patients who had other known abdominal pathology, prior large abdominal surgery, surgery involving the RYGB, or previous surgery for internal herniation were excluded from participation in the study. Subjects who had not undergone a diagnostic laparoscopy were excluded from the study, even after CT examination. Patients were informed about the study by the attending physician. Informed consent for participation was obtained by the investigators.

### Imaging Protocol

Imaging was performed using a clinical CT scanner (iCT 256, Philips Healthcare, Amsterdam, The Netherlands) following a standardized protocol. Patients were subjected to a biphasic abdominal CT examination (arterial phase CTA and portal venous CT). Neutral oral contrast medium was administered, consisting of 250 mL mannitol 10% (Baxter, Deerfield, Illinois, USA), 750 mL water and 3 scoops of Nutrilon Nutriton (Nutricia, Schiphol, The Netherlands). This composition provides intestinal distention without interfering with vascular enhancement, enabling the isolation of the mesenteric vasculature on the CTA. Volume and flow rate for IV contrast medium (Xenetix 300 mg I/mL, Guerbet, Villepinte, France) were stratified for body weight. In all subjects, 30 mL saline flush was administered. Using a standard bolus tracking technique, the CTA of the mesenteric arteries was attained, directly followed by the venous phase CT examination. Technical details on both scan protocols can be found in Table 1, intended for improving reproducibility of the described CT examinations by radiologists. Prior to scanning, patients were asked about current complaints that might be related to an active internal hernia.

**Table 1:** Imaging protocols

	Arterial CTA	Venous CT
Delay (s)^a^	15	65
Reconstruction algorithm	IMR level 2	iDose level 3
X-ray tube kV peak^b^	102 [100–120]	104 [100–120]
X-ray tube mAs^b^	196 [117–301]	164 [104–259]
IV contrast volume (mL) @ injection rate (mL/s)		
Body weight (kg)		
< 60	90 @ 3.5	
60–80	110 @ 4.0	
< 80	125 @ 4.5	
Acquisition FOV	Diaphragm dome to lesser trochanter	
Pixel size (mm × mm)^b^	0.70 × 0.70 [0.63 × 0.63–0.79 × 0.79]	
Slice thickness (mm)	0.90	
Reconstruction matrix	512 × 512	

*CTA* computed tomography angiography, *CT* computed tomography, *IMR* iterative model reconstruction, *IV* intravenous, *FOV* field of view

^a^Post-threshold delay in aorta at diaphragm level

^b^Average values, range between brackets

### Diagnostic Laparoscopy

During diagnostic laparoscopic surgery, all elements of the RYGB were evaluated following a standardized approach. Firstly, both potential hernia sites were inspected without manipulating the small intestine. The next step was to evaluate all three intestinal limbs and both blind limbs. The closure of the mesenteric defects was assessed, along with the presence of an active internal hernia. If present, the hernia was resolved and a complete closure of the mesenteric defects was achieved using non-absorbable V-Loc (Covidien, Medtronic, Dublin, Ireland). All findings were documented by the performing surgeon using a standardized form.

### Image Processing and Analysis

The venous phase CT examination was reconstructed using the Philips iDose algorithm. This examination was clinically assessed by a specialized radiologist, as is the norm. The CTA was reconstructed using the Philips iterative model reconstruction (IMR) algorithm for noise reduction. Using IntelliSpace Portal 8.0 (Philips Healthcare, Amsterdam, The Netherlands), a 3D reconstruction of the mesenteric arteries and surgical staples was obtained (3D CTA). The 3D CTA studies were retrospectively analyzed by a radiologist, a bariatric surgeon, and two bariatric researchers for potential signs of internal herniation. The observers of the 3D CTA studies were aware of the outcome of the diagnostic laparoscopy.

Patients in the study cohort were, dependent on the findings of the diagnostic laparoscopy, categorized as *control* (completely closed potential hernia sites upon laparoscopic inspection), *active internal herniation* (active internal hernia upon laparoscopic inspection and complaints during CT examination), or *inconclusive* (upon laparoscopic inspection, one or both mesenteric defects were open, yet no active internal hernia was found). In the latter group, both the presence and possible type of internal hernia during scan acquisition are indefinite. These cases are therefore not considered to be part of the study. 3D CTA studies of subjects with active internal hernias were compared with controls, in order to reveal signs of internal hernias. To gain a thorough understanding of the normal anatomy after RYGB surgery, four additional CTA examinations of post-RYGB patients were retrospectively analyzed and added to the control group. These were selected from a database of post-RYGB patients who had previously undergone a CTA in with normal findings.

## Results

### Cadaver Study

**Fig. 2:**
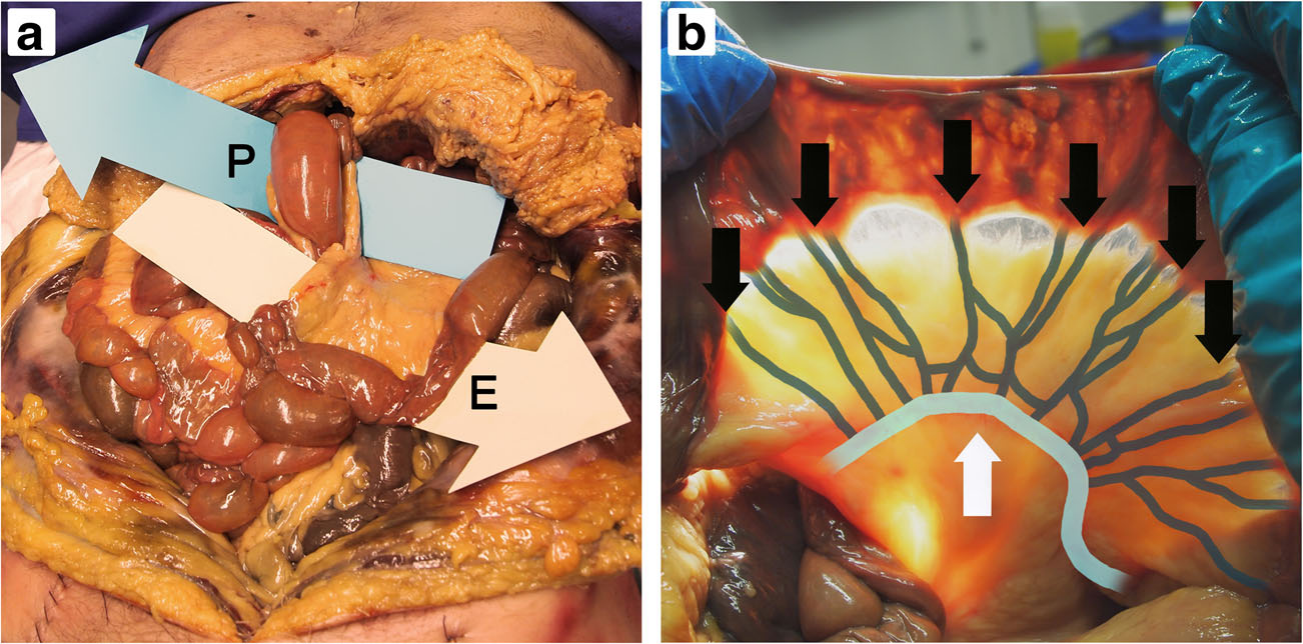
During cadaveric analysis, both potential hernia sites were clearly identifiable (**a**). Petersen’s defect (P) and the enteroenterostomy defect (E) are indicated. The SMA was recognized, along with the arterial arcade (**b**), emphasized by black and white arrows, respectively

**Fig. 3:**
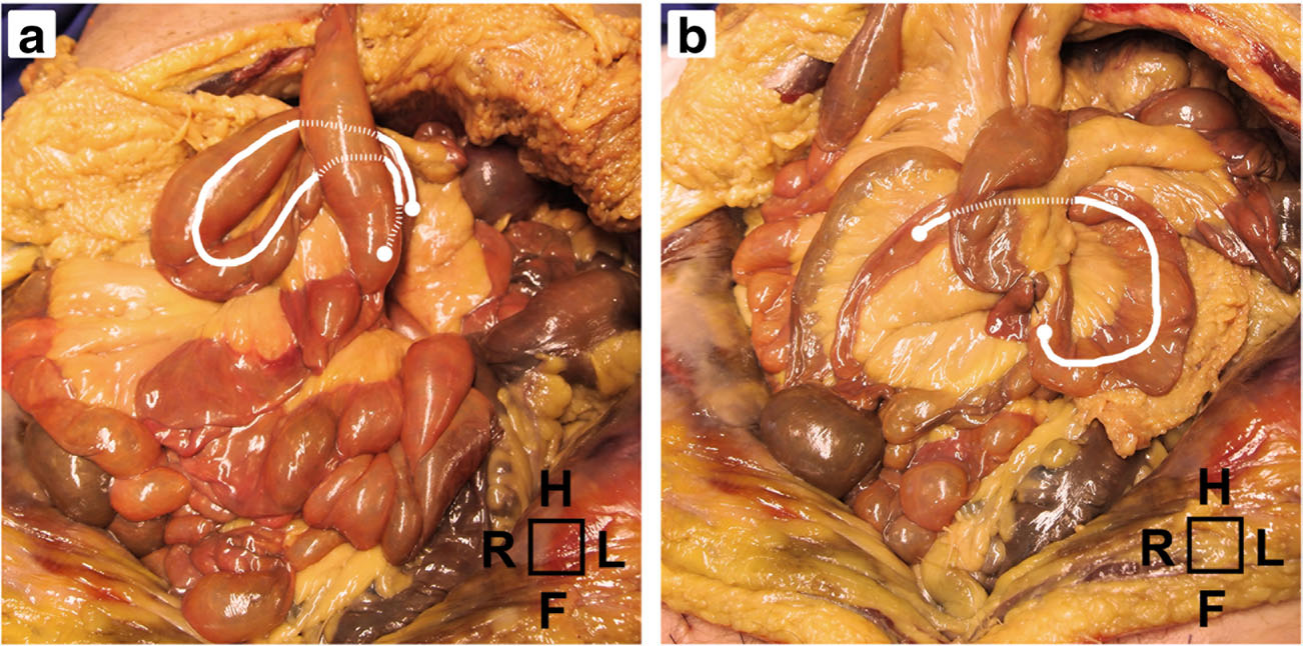
Simulations of two commonly diagnosed internal hernia types on a cadaver; biliopancreatic limb through Petersen’s defect from left to right (**a**) and common channel through enteroenterostomy defect from right to left (**b**). These images can be compared to the illustrations in Fig. 1

During cadaveric dissection, both potential hernia sites were clearly identifiable (Fig. 2a). All 12 basic hernia types were simulated on a cadaver (Supplemental Figure), although certain types seemed to be anatomically impossible to recreate. The alimentary limb through the enteroenterostomy defect (left-to-right) and the common channel through Petersen’s defect (left-to-right) were particularly difficult to simulate, due to traction on the mesentery. Two examples of commonly occurring internal hernias are shown in Fig. 3.

**Fig. 4:**
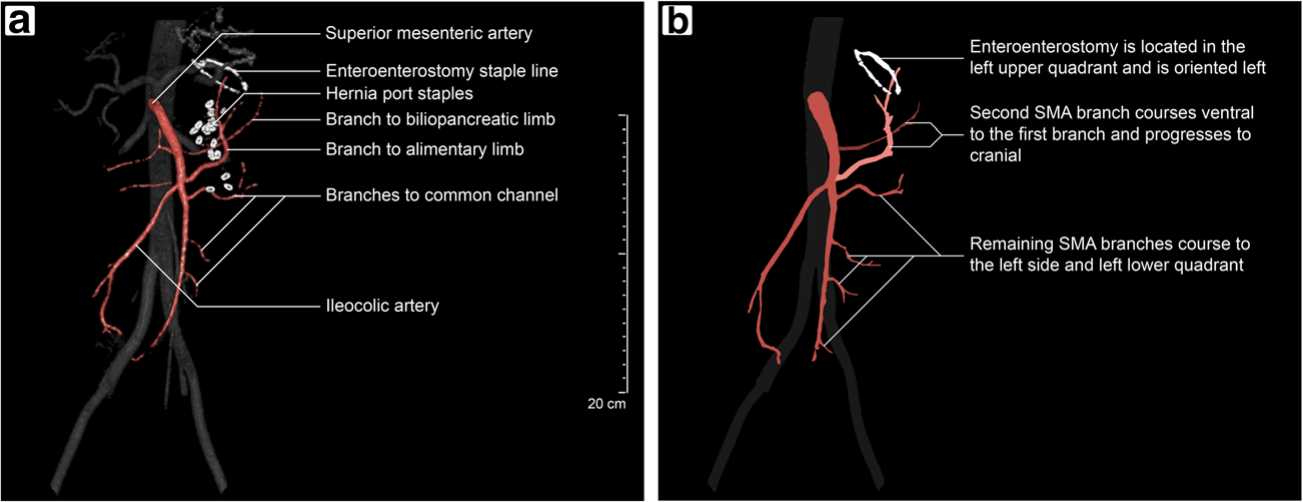
Relevant anatomical structures on control 3D CTA studies have been identified (**a**) and characteristics are displayed schematically (**b**). Both images employ an anterior view

The superior mesenteric artery (SMA) and the arterial arcade were also recognized (Fig. 2b). The mesenteric vascularization was investigated, both in the absence as well as the presence of an internal hernia. Examination confirmed that mesenteric swirl and migration of the mesenteric vasculature are often inevitable in the presence of an internal hernia. Based on the findings during cadaveric dissection, the following interpretations of control 3D CTA studies (Fig. 4a) are most evident:

- The first major SMA branch on the left supplies the biliopancreatic limb;
- The second major SMA branch on the left supplies the alimentary limb;
- Remaining major SMA branches on the left supply the common channel.

### Demographics and Main Outcomes of Clinical Study

Fifteen patients were initially included and subjected to a biphasic CT(A) examination. Five of these patients (study subjects 2, 4, 5, 8, and 11) were excluded because their complaints had suddenly ceased and therefore, laparoscopic exploration for internal herniation was not indicated. Patient demographics and characteristics of the ten resulting study subjects can be found in Table 2.

**Table 2:** Patient demographics and clinical characteristics

Study subject^a^	1	3	6	7	9	10	12	13	14	15	Mean
Gender	F	M	F	F	F	F	F	F	M	F	80% F
Age (year)	49	35	51	62	53	50	54	37	72	43	51
Pre-operative weight (kg)^b^	115	146	125	104	115	106	131	135	119	97	119
Pre-operative BMI (kg/m^2^)^b^	42	42	40	42	37	35	44	41	42	40	41
Weight at time of CT (kg)	58	88	105	77	70	72	96	99	102	52	82
BMI at time of CT (kg/m^2^)	21	25	34	31	23	24	32	31	37	21	28
Clinical presentation											
Intermittent abdominal pain	+	+	+	+	+	+	+	+	+	+	100%
Postprandial abdominal pain	–	–	+	+	+	–	–	+	+	+	60%
Nausea	–	–	+	–	+	+	–	–	+	+	50%
Vomiting	–	–	–	–	–	–	–	–	+	+	20%
Inclusion via	OC	OC	OC	OC	OC	OC	OC	OC	ED	OC	90% OC
Time from primary surgery to CT (weeks)	112	54	289	53	262	120	92	159	297	67	151
Time from CT to DL (weeks)	16	1	11	10	6	16	6	3	0	5	7

*BMI* body mass index, *CT* computed tomography, *OC* outpatient clinic, *ED* emergency department, *DL* diagnostic laparoscopy

^a^Data of study subjects who were not subjected to diagnostic laparoscopy is not included

^b^Prior to primary RYGB surgery

In three cases, both of the mesenteric defects were found to be completely closed upon diagnostic laparoscopy. With the addition of four retrospective cases, this resulted in seven controls. In three patients, an active internal hernia was found during laparoscopic exploration. In the remaining four subjects, one or both mesenteric defects were open, yet no active internal hernia was found.

One postoperative complication was reported (study subject 7); a trocar wound retraction, for which exploration on the outpatient operating room was indicated. Abdominal complaints did not resolve immediately after diagnostic laparoscopy in this subject, but were resolved in both other subjects with active internal hernias. Abdominal complaints in control subjects were attributed to omental adhesions, excessive blind limb length or unknown causes. All patients were discharged on the first postoperative day. Scan and surgery outcomes are denoted in Table 3.

**Table 3:** Scan and surgical characteristics

Study subject^a^	1	3	6	7	9	10	12	13	14	15
Abdominal complaints at time of CT	+	+	+	+	+	–	–	–	+	+
IH diagnosed on CT^b^	–	–	–	–	–	–	–	–	+	–
Potential hernia sites primarily closed	+	+	–	+	+	+	+	+	–	+
Potential hernia sites closed upon DL^c^	–	+	+	–	–	+	–	–	–	–
Involved defect (size (cm))	ED (5)			PD (5)	PD (5)		PD (3)	PD (1)	PD (5)	PD (N/A)
					AD (5)^d^		ED (6)	ED (5)	ED (10)	ED (N/A)
Active IH upon DL	+	–	–	+	–	–	–	–	+	–
Involved defect	ED			PD					ED	
Involved intestinal limb	CC			BL					AL + ES	

*CT* computed tomography, *DL* diagnostic laparoscopy, *IH* internal herniation, *ED* enteroenterostomy defect, *PD* Petersen’s defect, *CC* common channel, *BL* biliopancreatic limb, *AL* alimentary limb, *ES* enteroenterostomy, *N/A* not available

^a^Data of study subjects who were not subjected to diagnostic laparoscopy is not included

^b^As clinically assessed by a radiologist

^c^Upon initial inspection, before surgical closure

^d^AD = aberrant defect; at the enteroenterostomy, an additional potential hernia site had been formed

### Control 3D CTA

To give an impression, a 360° view of a control 3D CTA study (study subject 3) is included (Supplemental Video). Relevant anatomical structures on control 3D CTA studies are highlighted in Fig. 4a. Characteristic findings on control 3D CTA studies (Fig. 4b), occurring at least six out of seven times in all control cases, include the following:

- The enteroenterostomy staple line is located in the left upper quadrant;
- The opening of the enteroenterostomy staple line has rotated towards the left;
- The second left branch of the SMA courses ventral to the first branch and progresses to cranial;
- Remaining left SMA branches predominantly progress to the left side and left lower quadrant.

### 3D CTA and Internal Hernias

**Fig. 5:**
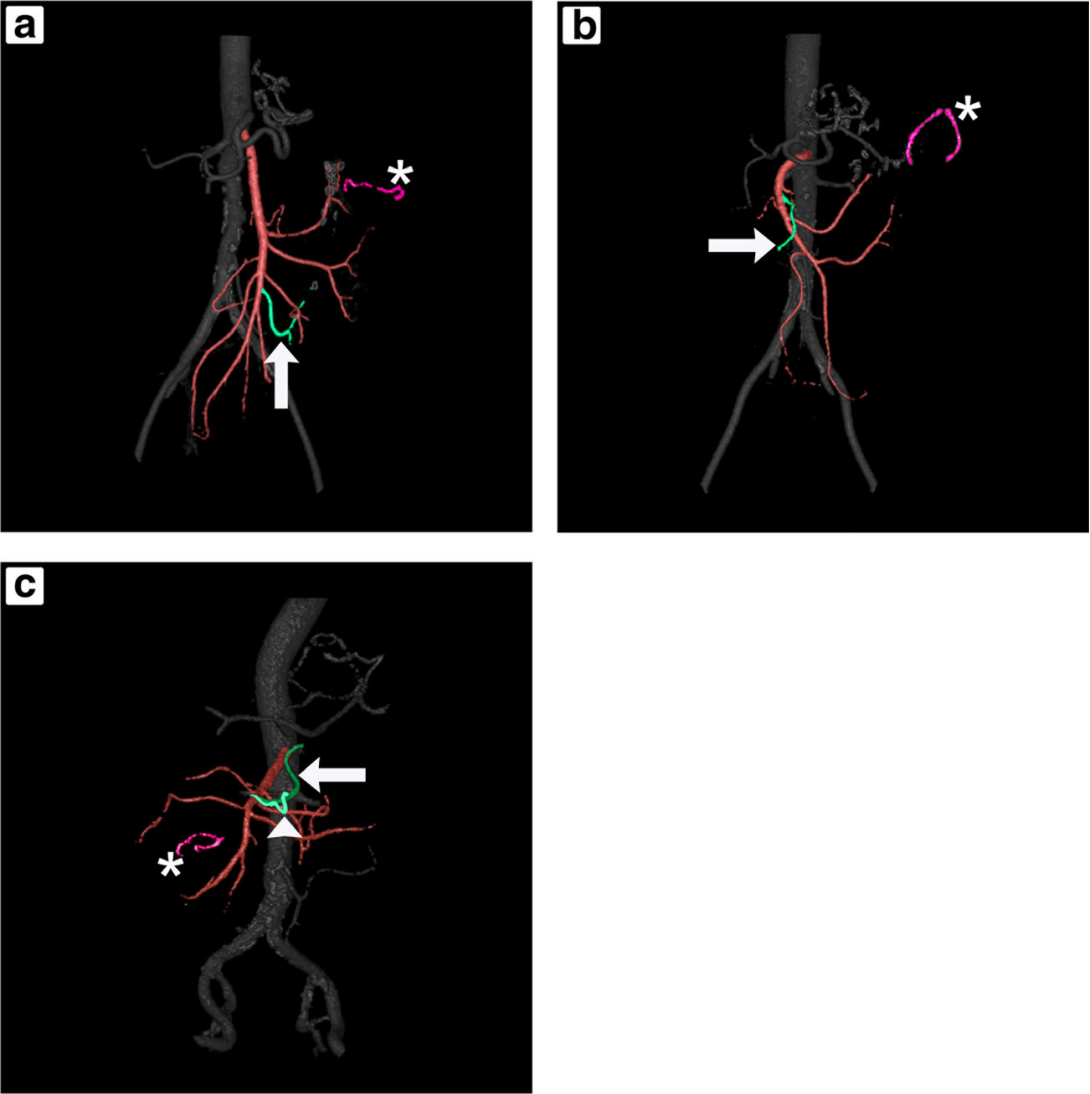
Anterior view of 3D CTA studies with presumed active internal hernias. Three hernia types were identified; common channel through enteroenterostomy defect (**a**), biliopancreatic limb through Petersen’s defect (**b**), and alimentary limb with enteroenterostomy through enteroenterostomy defect (**c**). Remarkable SMA branch configurations and enteroenterostomy staple line arrangements are indicated by an arrow(head) and an asterisk, respectively

Figure 5 shows the 3D CTA studies of three patients (study subjects 1, 7, and 14) with presumed active internal hernias. Three internal hernia types were identified upon laparoscopic exploration; common channel herniated through the enteroenterostomy defect (Fig. 5a), biliopancreatic limb herniated through Petersen’s defect (Fig. 5b) and alimentary limb with enteroenterostomy herniated through the enteroenterostomy defect (Fig. 5c). Only the last case was diagnosed on venous phase CT examination by a specialized bariatric radiologist. All three subjects had complaints shortly before scan acquisition, making an active internal hernia at that time plausible.

Remarkable differences can be observed when compared to controls (Fig. 4). The first case (Fig. 5a) shows an SMA branch, corresponding to the common channel, coursing to *cranial* instead of the left lower quadrant. Furthermore, the opening of the enteroenterostomy staple line has rotated towards *dorsal* instead of left. In the second case (Fig. 5b), the first left SMA branch, corresponding to the biliopancreatic limb, courses *ventral* to the second branch and progresses to the *right.* In this case, the opening of the enteroenterostomy staple line has also rotated towards *dorsal*. The third case (Fig. 5c) shows a remarkable trajectory of the second SMA branch, which supplies the alimentary limb. This branch entirely courses to *ventral* (arrow), while a division of this branch progresses to the *right* (arrowhead). Additionally, this case shows a right-sided enteroenterostomy staple line.

## Discussion

After many years, abdominal venous phase CT examination is still the first-line imaging technique for diagnosing internal herniation after RYGB surgery [9, 18]. However, diagnosing internal hernias accurately remains a challenge, for many reasons. First and foremost, the appearance of the small intestine varies over time and between individuals, especially due to its mobility. A complicating factor is the anatomical alterations as a consequence of RYGB surgery. On CT examinations, the trajectory of the small intestine may be difficult to assess due to incomplete filling of the intestinal lumen with contrast agent. To make things more complicated, clinical CT examination may depend primarily on 2D, rather than 3D assessment, thus impeding the spatial inspection of anatomical structures. Furthermore, internal hernias may be intermittent or self-resolving, making image acquisition at time of symptomatology crucial. Additionally, more than 12 different configurations of internal hernias have been identified, both theoretically and experimentally. Hence, *the one defining* internal hernia, as it is often presented, does not exist.

Although internal herniation may be diagnosed by exclusion of other pathology on CT examinations, the detection of internal herniation *itself* is desirable. When internal herniation can be diagnosed with greater confidence, the decision of whether a patient should undergo laparoscopic surgery can also be made with greater determination. A wide variety of CT signs have therefore been proposed for diagnosis of internal herniation [9–14]. It has been demonstrated repeatedly that the swirl sign is the best predictor for internal herniation. On average, the sensitivity of the swirl sign is 75% (39–100%) and the specificity 85% (63–100%) [9–14]. Cadaveric dissection confirmed that mesenteric swirl is often inevitable in the presence of an internal hernia, hence confirming the predictive value of this sign. Other classic CT signs, such as the mushroom sign and hurricane eye, are highly specific (usually 90%), but lack sensitivity (typically 35%) [9–14]. These signs may be focused disproportionately on the intestine alone, or depend excessively on 2D assessment.

There has been a sustained effort put into the development of alternatives that will be able to address these issues. Up to now, alternative CT signs that rely less on the assessment of the intestines have not yielded the desired result. Signs such as engorged lymph nodes, ascites, dilated gastric remnant, or edema have a specificity of around 90%, but a limited sensitivity of about 40%; comparable to most classic CT signs [10–12, 14]. Recently, it has been shown that a decision tree model using several CT signs, including newly proposed signs, may improve diagnostic accuracy [9]. As the authors have stated, further prospective research should prove the clinical implication of this conclusion. Another interesting possibility is to assess the small intestine on 3D reconstructions of intraluminal air and contrast medium. However, manual segmentation of the small intestine on CT examinations is time-consuming and labor-intensive. It has been reported, however, that automatic volume rendering of the small intestine may aid in diagnosing internal hernias [16]. Still, this does not resolve the complexity of assessing the intestine due to incomplete intraluminal filling with contrast agent or due to its variable trajectory. Hence, 3D reconstructions of the small intestine, be it manually or automatically generated, may not become clinically applicable.

In a recent report, the use of 3D reconstruction of the mesenteric vasculature for diagnosing non-RYGB-related internal hernias was mentioned [16]. The present study, however, is the first to report the use of 3D CTA for diagnosing internal herniation *after RYGB surgery*. Moreover, the features on normal 3D CTA after RYGB surgery were established and confirmed by cadaveric dissection. In control cases, the second SMA branch on the left demonstrates a remarkable course with respect to the first branch. Nonetheless, this is an expected finding after RYGB surgery: the proximal segment of the jejunum (biliopancreatic limb) is almost untouched, while the distal segment (alimentary limb) is brought up. The ileocolic artery normally marks the transition between the blood supply of the jejunum and the ileum [19, 20]. After RYGB surgery, this can be roughly translated as the transition between the blood supply of the alimentary limb and the common channel. We have demonstrated that 3D CTA studies with active internal hernias display notable differences in mesenteric vasculature and surgical staple arrangement. In all study subjects with active internal hernias, the changes in vascular structure corresponded to the migration of herniated intestinal limbs.

The enteroenterostomy staple lines on control 3D CTA studies were located in the left upper quadrant and had rotated towards the left. This was an expected finding, based on how the RYGB procedure is performed. In all subjects with active internal hernias, the enteroenterostomy staple lines changed orientation. In the presence of an internal hernia, herniated intestinal limbs may cause tension on the enteroenterostomy. Thus, a rotation of the enteroenterostomy staple line seems a logical response. Migration of the enteroenterostomy has been reported before; a right-sided staple line is one of the classic CT signs for internal herniation [9–11, 13, 14]. This sign has a reported sensitivity of 0–22% (on average 11%), although it is highly specific (on average 97%, ranging from 90 to 100%) [9–11, 13, 14]. The lack of sensitivity is explained by the fact that migration of the enteroenterostomy is absent in most types of internal hernias. Nevertheless, the change in *orientation* of staple lines is potentially a novel diagnostic feature.

These results demonstrate that 3D CTA is a promising technique for diagnosing internal herniation after RYGB surgery. Our findings imply that for diagnosing internal hernias, focus should probably shift towards 3D assessment of CT examinations, and specifically to 3D assessment of the mesenteric vasculature and surgical staples. 3D CTA allows for examination of the intestinal blood supply, is readily available on other radiological workstations and clinically applicable. After basic training, 3D reconstruction of CTA examinations is achievable within minutes. Since 3D CTA is based on 2D CTA, conventional 2D assessment is still possible to rule out other pathology, and both are advocated.

Novel techniques may also aid in the *prevention* of internal herniation, rather than improving diagnostic accuracy. A well-known example is the primary closure of the mesenteric defects, for which various methods have been proposed. Primary closure could have prevented internal herniation in the two (out of ten) patients in this study, in whom the defects were not primarily closed. Recent literature suggests that closure with a running suture is superior to stapled closure [6, 7]. However, results for stapled closure in two layers, the surgical technique employed at our center, are absent. Regardless of the technique used for primary closure, the incidence of internal hernias has not yet been decreased to 0%, unfortunately.

Our study has several limitations worth noting. Firstly, this is a small series of ten study subjects, which impedes the differentiation between anatomical variants and true signs of internal hernias. We believe further study should establish a solid description of control characteristics, as well as true diagnostic features. Secondly, it was assumed that patients with an active internal hernia during diagnostic laparoscopy also had an active hernia during scan acquisition. We anticipated on this limitation by documenting abdominal complaints prior to scan acquisition; all patients with presumed internal hernias had complaints at that time. Finally, the surgical outcome was known by the observers when examining 3D CTA studies for signs of internal herniation. While this may result in “cherry picking” evidence for internal herniation, this is justified by the fact that the present study was the first of its kind. The added value of 3D CTA should be confirmed objectively by means of a blinded comparative study between the proposed method and standard CT examination. As from the end of this study, all patients with suspected internal herniation are examined according to the altered CT protocol in our institution. The data will be used for further investigation of the newly developed diagnostic method. In further research, the described method could be explored in other clinical situations where internal hernias occur, such as after gastrectomy or colorectal resections. It is essential, however, to fully understand the normal anatomy and anatomical variants in such patient groups, before attempting to diagnose pathological conditions.

## Conclusion

In conclusion, the present study outlines and demonstrates the complexity of diagnosing internal hernias after RYGB surgery by means of a literature overview, cadaveric dissection, and a clinical study. This study shows that 3D CTA is a promising technique for diagnosing internal herniation, albeit on a small number of subjects. 3D CTA studies of subjects with active internal hernias demonstrated alterations in the trajectory of the blood supply of herniated intestinal limbs. In addition, enteroenterostomy staple lines were migrated or changed orientation in the presence of an internal hernia. The presented findings imply that for diagnosing internal hernias, more effort should be directed towards the 3D assessment of the mesenteric vasculature and surgical staples. It is our presumption that further study will confirm the added value of the proposed method, but before introducing it as a standard diagnostic tool, further validation is necessary.

## Electronic Supplementary Material

ESM 1 All twelve basic internal hernia types, simulated on a cadaveric abdomen. Herniated limb segments are highlighted in white. Hernia types with an asterisk (*) seemed anatomically impossible. H = head, F = feet, R = right, L = left. (JPEG 1649 kb)

High resolution image (TIFF 2164 kb)

ESM 2 360° view of a control 3D CTA subject (study subject 3). Note the position and orientation of the mesenteric arteries and enteroenterostomy staple line. (MP4 842 kb)
