# Supplementary figures and images for: Diagnosing Internal Herniation After Roux-en-Y Gastric Bypass Surgery: Literature Overview, Cadaver Study and the Added Value of 3D CT Angiography
Source: Obes Surg. 2018 Feb 5;28(7):1822–30. doi: 10.1007/s11695-018-3121-3 (PMC6107799; doi:10.1007/s11695-018-3121-3)

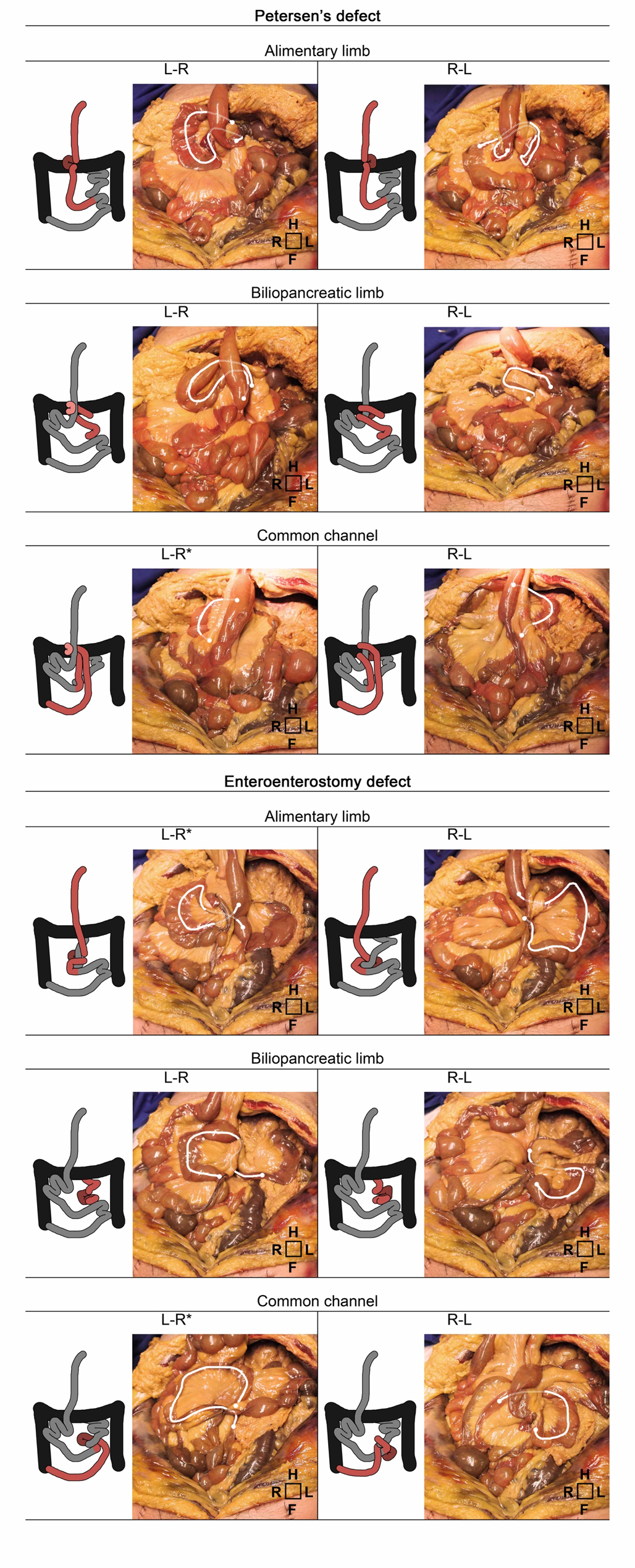

Supplement: Supplementary file 1 — All twelve basic internal hernia types, simulated on a cadaveric abdomen. Herniated limb segments are highlighted in white. Hernia types with an asterisk (*) seemed anatomically impossible. H = head, F = feet, R = right, L = left. (JPEG 1649 kb) [file 11695_2018_3121_Fig6_ESM.jpg]
